# Medical Students' Preparedness to Discuss Lesbian, Gay, Bisexual, Transgender, Queer, and Other (LGBTQ+) Patient-Related Topics: A Survey Study

**DOI:** 10.7759/cureus.34237

**Published:** 2023-01-26

**Authors:** Matthew S Khaleghi, Karen Andrawes, Jordan Yacoub, David Kashmer

**Affiliations:** 1 Simulation, Edward Via College of Osteopathic Medicine, Auburn, USA

**Keywords:** discrimination in health care, simulation research, simulation in medical education, lgbtq+, lgbtq

## Abstract

How well do doctors know their patients? Is the future generation of doctors prepared for real-world patient encounters? Lesbian, gay, bisexual, transgender, queer, and other (LGBTQ+) patients are disproportionately affected by a wide range of health issues, and many of these patients face barriers and stigma when accessing healthcare. Our research aimed to explore the awareness current medical students hold toward some of the health disparities faced by LGBTQ+ patients. Second-year medical students at our institution filled out a survey following their standardized patient exams to examine how prepared they felt to diagnose and treat a patient self-identifying as a member of the LGBTQ+ community.

## Introduction

According to a 2021 poll by GALLUP, 5.6% of Americans self-identify as members of the lesbian, gay, bisexual, and transgender (LGBT) community [1]. However, more than 50% of LGBT people experience some form of healthcare discrimination [2]. This discrimination raises suspicion of whether doctors in healthcare today are competent in discussing lesbian, gay, bisexual, transgender, queer, and other (LGBTQ+)-related health services with their patients. We sought to see if current medical students feel adequately prepared to discuss some of these issues during their standardized patient exams. Second-year medical students were asked to fill out a survey following their standardized patient exams to see how prepared they felt to diagnose and treat a standardized patient self-identifying as a member of the LGBTQ+ community. This survey study opens up possibilities for future curricular intervention to compare students' preparedness [3,4]. A discussion among professionals and community members determined the prevalent disparities faced by the LGBTQ+ community and the benefit of implementing educational opportunities to foster positive healthcare counseling in a professional setting [5]. It is suggested that to minimize negative healthcare experiences of LGBTQ+ patients, correct terminology, preferred names and pronouns, and obtaining unbiased and unassuming histories are necessary for increasing preparedness for the care of individuals of sexual minorities [6]. Studies have shown that there is a significant importance in healthcare providers knowing the sexual identity and gender identity of their patients; hence they can counsel appropriately about topics including human immunodeficiency virus (HIV) and sexually transmitted infection testing, behavioral health education, and cervical cancer screenings [7]. Additionally, studies have revealed that providers do not always screen bisexual women according to updated United States Preventive Services Task Force (USPSTF) guidelines due to a lack of understanding of the female patient's sexual history [7]. Women of sexual minority groups have higher rates of depression paired with a greater risk of risky coping mechanisms, yet there is a lack of counseling tools for healthcare providers tailored to account for the stressors placed on that specific group [8,9]. A lack of fundamental education can be blamed for a sizable portion of such disproportion. Clinicians, their patients, healthcare administrators, and insurance providers would clearly benefit from a higher level of competence and confidence when caring for individuals in the LGBTQ+ community. Teaching and education are the best ways to tackle issues and introduce LGBTQ+ health into a school's curriculum. Our study, among others, demonstrates the need to prioritize additional research and implementation of more extensive medical training regarding sexual orientation and gender identification in order to overcome barriers to sufficient care for LGBTQ+ individuals [10]. This article was previously presented as a poster at the Fifth Annual Via Research Recognition Day on February 18, 2022.

## Materials and methods

Data were collected from all second-year medical students participating in the standardized patient exam on 12/7/2021 in Auburn, AL. We believed that collecting data from the entire student population at our institution would generate the most representative sample to study (n = 153). The curriculum at all Edward Via College of Osteopathic Medicine (VCOM) campuses consists of preclinical education in the first two years, with simulated clinical encounters in each concentrated block of study. Second-year students were selected to study since they had already encountered most of the institution's didactic instruction and had the greatest chance of previously being exposed to LGBTQ+ matters in healthcare. To assess whether our students were comfortable discussing LGBTQ+ patient-related topics in their clinical encounters, we asked them to fill out a simple survey.

An anonymous paper survey was given to students containing one question: “Do you feel adequately prepared to discuss LGBTQ+-related topics in healthcare today?” with a yes or no checkbox. Response (1) was “yes.” If the student selected “no”, there was a list of six boxes to check for potential reasons why they chose no. The five checkboxes were as follows: (2) I have not received adequate education regarding LGBTQ+-related matters, (3) I have received adequate education regarding LGBTQ+ matters but am not confident in applying it clinically, (4) I have received adequate education regarding LGBTQ+ matters, but I have not had a chance to apply it clinically, (5) I believe LGBTQ+ issues in healthcare are not much different than heterosexual issues, (6) I am not comfortable discussing LGBTQ+-related matters, and (7) Other. Participants filled out their paper surveys at their individual computer stations. After exiting their clinical encounters, surveys were placed in opaque collection bins to ensure confidentiality. All students and standardized patients followed the VCOM-Auburn coronavirus disease 2019 (COVID-19) health protocol.

## Results

**Figure 1:**
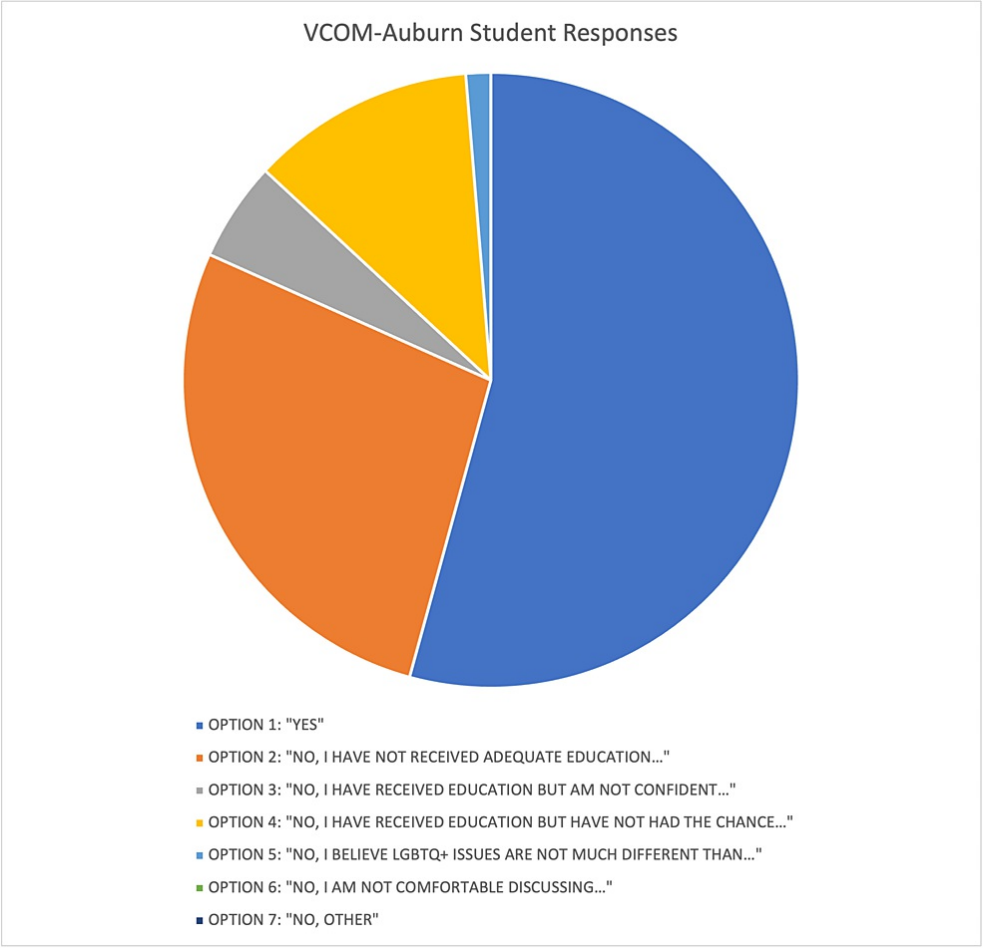
VCOM-Auburn Student Responses Data from the paper surveys given on 12/7/2021 immediately following student standardized patient exams.

Results revealed a total of 153 responses from the students: 83 for response one, 42 for response two, eight for response three, 18 for response four, two for response five, and zero responses for both options six and seven. Overall, 54% of medical students said they felt adequately prepared to discuss LGBTQ+ matters in a clinical setting, whereas 46% said they did not (Figure 1). The most common reason students selected that they did not feel prepared for an encounter with an LGBTQ+ patient was a lack of adequate education (60% of “no” responses).

## Discussion

This survey study found that 54% of medical students at our institution believed that they have had adequate education regarding LGBTQ+ matters in their curriculum thus far. Most of the remaining students reported that more education and exposure to LGBTQ+ health training would benefit their learning. Specific training found within the medical curriculum is associated with better knowledge and awareness of LGBTQ+ health issues and disparities [3]. Results suggested a preference for increased teaching and implementation of LGBTQ+ issues in the course of the study. Curriculum changes can be approached in many ways to disperse content and add to existing lectures. The information could also be given in topic-specific discussions per specialty topic. Institutional culture can be altered to include more LGBTQ+ health content if students work diligently with their faculty and administration [4]. Students currently receive didactic lectures on various topics during clinical rotations and relating topics could easily be implemented to overcome the lack of faculty expertise and time to incorporate this into an existing preclinical curriculum [8]. Ongoing collaborations with professors and student leaders alike would prove to aid curriculum deficiencies. Interventions can also be made to improve LGBTQ+ healthcare training. These interventions can address matters related to LGBTQ+ health disparities, including mental health struggles, discrimination when accessing care, and increased risk of substance abuse. Workshop events could also be introduced at host institutions, in which experienced students and faculty can teach evidence-based approaches to communication and rapport building with LGBTQ+ individuals. Other ways that LGBTQ+-specific disparities could be addressed throughout medical education are either the development of an elective course or by incorporating topic-specific cases into a school's simulation center through skill-based laboratory time or standardized patient experiences [8]. Such cases allow a firsthand experience of interacting with these patients and give medical students time to practice the essential skills needed when communicating with patients of special populations. Most current research on barriers to care of LGBTQ+ individuals consists of self-reported data and surveys, rather than direct methodology [10]. With a continued study involving an intervention, it is expected to gather better data to build on current literature. Further time and funding are expected to be directed toward projects confronting barriers to care. Addressing provider competence through education toward LGBTQ+ issues is only the first step in tackling barriers. Further gaps in knowledge to be addressed in the near future should involve stereotypes, biases, cultural norms, socioeconomics, and health system barriers.

## Conclusions

In conclusion, marginally greater than one half of second-year medical students at VCOM-Auburn answered "yes" on our study questionnaire, indicating they felt adequately prepared to discuss LGBTQ+ matters in a clinical setting. From the remaining "no" responses, the most frequent response marked by students was for the option suggesting a lack of adequate education regarding LGBTQ+ related matters. Future investigation into an education intervention could improve the number of “yes” responses and lead to better outcomes following simulation department examinations. The didactic method of teaching during the first two years of medical school serves as the primary way of introducing students to LGBTQ+ topics in healthcare. Simulation is the best way for students at our institution to approach actively discussing LGBTQ+ related matters in a clinical setting. We hope that this project leads to quality improvement within the simulation department, followed by curriculum changes in the institutional course of study. Through continued exposure to unique patient populations, students can feel more adequately prepared to discuss LGBTQ+-related topics in healthcare. As future physicians, these skills will prove to be beneficial in creating healthy patient-physician relationships, in which their future patients feel cared for and respected as individuals.
